# The relationship of leukoaraiosis, haemorrhagic transformation and prognosis at 3 months after intravenous thrombolysis in elderly patients aged ≥ 60 years with acute cerebral infarction

**DOI:** 10.1007/s10072-020-04398-2

**Published:** 2020-05-01

**Authors:** Xiaowei Liu, Jiatang Zhang, Chenglin Tian, Jun Wang

**Affiliations:** 1 Medical School of Chinese PLA, Medical School of Chinese PLA, Beijing, China; 2 Chinese PLA General Hospital, No. 28, Fuxing Road, Beijing, 100853 China; 3 Beijing Huairou Hospital, No.9, Yongtai North Street, Beijing, 101400 China

**Keywords:** Leukoaraiosis, Haemorrhagic transformation, Prognosis, Intravenous thrombolytic, Ischemic stroke, Geriatric

## Abstract

**Backgroud:**

As the elderly stroke population continues to increase, we will have to confront greater challenges regarding how to choose suitable patients to reduce thrombolysis-related bleeding events and accurately judge their prognosis. Therefore, we evaluated the relationship among leukoaraiosis (LA), haemorrhagic transformation (HT) and the prognosis at 3 months after intravenous (IV) thrombolysis in elderly patients aged ≥ 60 years with acute cerebral infarction (ACI).

**Methods:**

We prospectively and consecutively chose 125 elderly patients aged ≥ 60 years with ACI who could accept and be suitable for IV recombinant tissue plasminogen activator (rtPA) after excluding 6 cases. Brain computed tomography(CT) was used to assess LA by using the modified Van Swieten scale (mVSS) before treatment and the modified Rankin scale (mRS) to appraise prognosis at 3 months after IV rtPA. Binary logistic regression was used to analyse the predictors of HT and the prognosis of ACI.

**Results:**

Our data indicated that by brain CT, 26.4% of all patients showed severe LA, and the rate of HT and symptomatic intracranial haemorrhage (sICH) were 12.0% and 9.6%, respectively. Severe LA was evidently associated with HT (odds ratio [OR] 3.272, 95% confidence interval [CI] 1.010–10.598, *P* = 0.048) rather sICH (*P* > 0.05). Moreover, we also found that severe LA was associated with poor functional prognosis (OR 5.266, 95% CI 1.592–17.419, *P* = 0.006).

**Conclusion:**

Our results showed that LA was associated with HT and adverse clinical prognosis rather sICH after IV rtPA in elderly patients aged ≥60 years with ACI. Although LA may increase the risk of bleeding but not fatal haemorrhage after IV thrombolysis, therefore, we should actively select an appropriate elderly population for thrombolytic treatment and have reasonable judgments on the outcomes.

## Introduction

It is well-known that IV thrombolysis in the hyperacute phase with rtPA recommended according to current guidelines [1] is one of the most effective ways to treat ACI, which can reduce the stroke-related disability and mortality. However, this treatment has some serious complications, such as HT even fatal sICH [2], which can affect the clinicians’ treatment decisions. Whether LA will increase the risks of HT and adverse prognoses after IV rtPA is still controversial [3–6]. As the life expectancy of human beings continues to gradually increase, so too does the number of elderly patients with ACI [7]. Fortunately, elderly ACI patients can benefit from thrombolytic therapy [8]. However, LA is more common in geriatric populations than in young people [9]. Whether the risk of HT in elderly patients with LA increases after IV rtPA is uncertain, and the prognosis of these patients at 3 months also needs to be further assessed. The purpose of the present study was to better understand the above relationships in elderly patients aged ≥ 60 years with ACI.

## Methods

From January 2016 to December 2018, 131 patients with ACI who were aged ≥ 60 years and were to be treated with IV rtPA within 4.5 h after stroke attacked were continuously selected in the Huairou District of Beijing. The inclusion and exclusion criteria of National Institute of Neurological Disorders and Stroke (NINDS) in 1995 were consulted [2], but no upper age limit was applied. Basic demographic characteristics and clinical history (including hypertension, diabetes mellitus, pre-stroke**,** coronary heart disease(CHD), congestive heart failure(CHF), atrial fibrillation(AF), smoking, hyperlipidaemia, chronic kidney disease(CKD), pre-anticoagulate/antiplatelet therapy(Pre-AC/AP)) were recorded. Before IV rtPA, National Institutes of Health Stroke Scale (NIHSS) were assessed by two senior neurologists simultaneously; in the cases of inconsistency, a superior neurologist adjudicated. At 3 months after thrombolytic therapy, two other senior neurologists without any knowledge of the clinical information evaluated the patients’ functional prognosis by face-to-face or telephone interview. MRS scores were used to grade the functional prognosis, where a score of 0–1 points was considered good, and a score of 2 or more points was considered bad. All of the selected patients were given rtPA (0.6–0.9 g/kg according to the standard dose, with 10% of the dose bolus injected within 1 min and the remaining 90% pumped over the course of an hour). All patients were routinely examined by brain CT at 24 h except one dead of sICH within 1 h after treatment, and 91 patients were examined by magnetic resonance imaging (MRI) within 36 h after thrombolysis, and those whose symptoms deteriorated or who had suspicious bleeding were checked by CT at any time to exclude intracranial haemorrhage. HT was evaluated according to the European Co-operative Acute Stroke Study-II (ECASSII) criteria [10]. The images of the HT were read by two other senior radiologists who were completely blinded to the clinical information. If there was disagreement, more experienced radiologists were called in to make a decision. MVSS [11] was used to classify white matter lesions. Scores between 1 and 4 were classified as mild lesions, and scores greater than 4 scores were classified as severe lesions. The mVSS score was graded by two other senior radiologists who were fully blind to patients. In cases of disagreements, a more senior radiologist was called in to resolve the assessment. All patients signed informed consent and were approved by the Ethics Committee.

## Statistics

All data were analysed by using the SPSS 17.0 software package (SPSS Inc. Chicago, IL, USA). Measured data was assessed by ANOVA or a T test, and numeration data was assessed by a Chi-squared test. The relationships between variables were analysed by correlation and logistic regression analysis. *P* < 0.05 was considered significant.

## Results

A total of 131 eligible patients were enrolled, 6 cases of which were lost due to transfer or abandonment of treatment (5 cases in the non-mild group and 1 case in the severe group). The mean age was 73.2 years. The basic information is shown in Table 1. There were 43 patients without white matter lesions (34.4%), 49 patients with mild white matter lesions (39.2%) and 33 patients with severe white matter lesions (26.4%). According to the severity of the white matter lesions, the patients were divided into two groups (the non-mild lesion group and the severe lesion group). The baseline data of the two groups were not significantly different except for age and NIHSS score before thrombolysis (*P* = 0.02 and 0.02, respectively), as shown in Table 2. There were 15 patients (12.0%, 15/125) with haemorrhagic transformation after IV, including 12 cases (9.6%, 12/125) of sICH. Correlation analysis indicated that HT was related to LA, NIHSS score (severity of stroke) and congestive heart failure (CHF). After adjusting for confounding factors, binary logistic analysis indicated that LA were associated with HT and NIHSS (NIHSS scores ≥ 12 means severity), (*P* = 0.048, OR 3.272, 95% CI 1.010–10.598; *P* = 0.012, OR 5.042, 95% CI 1.434–17.272, respectively), as shown in Table 3. There were 47 cases with mRS scores < 2 and 45 cases with scores ≥ 2 at 3 months after IV in the non-mild LA group. In the severe LA group, there were 5 cases with mRS scores < 2 and 28 cases with scores ≥ 2. Binary logistic analysis showed that mRS score > 2 was related to the severity of stroke (*P* < 0.01; OR 9.594, 95% CI 3.133–29.378) and LA (*P* = 0.006; OR 5.266, 95% CI 1.592–17.419), as shown in Table 4.

**Table 1:** Baseline characteristics

All patients	*n* = 125, *N* (%)
Age(mean)	73.2 ± 8.2
Sex(female)	41(32.8)
NIHSS	10.5(2–38)
Hypertension	79(63.2)
Diabetes	28(22.4)
Pre-stroke	37(29.6)
CHD	53(42.4)
CHF	19(15.2)
AF	29(23.2)
Cigar	48(38.4)
Hyperlipidaemia	30(24)
CKD	4(3.2)
Pre-AC/AP	44(35.2)
LA	
0	43(34.4)
1–4	49(39.2)
5–8	33(26.4)

**Table 2:** Non-mild LA and severe LA groups baseline

	Non-mild	Severe	*P* value
Sex(male)	63	21	0.611
Age	71.8 ± 8.3	76.9 ± 7.5	0.02^a^
NIHSS	9.6 ± 7.7	13.2 ± 7.5	0.024^b^
Hypertension	55	24	0.186
Diabetes	20	8	0.767
Pre-stroke	28	9	0.733
CHD	36	17	0.217
CHF	12	7	0.262
AF	18	11	0.108
Cigar	35	13	0.891
Hyperlipidaemia	20	10	0.323
CKD	3	1	1.000
Pre-AC/AP	32	12	0.870

a and b have statistical significance

**Table 3:** Factors related toHT

	OR	95%CI	*P* value
NIHSS	5.042	1.434–17.727	0.012^a^
CHF	0.456	0.121–1.723	0.247
LA	3.272	1.010–10.598	0.048^b^

a and b have statistical significance

**Table 4:** Factors related to prognosis

	OR	95%CI	*P* value
Age	1.026	0.967–1.089	0.394
NIHSS	9.594	3.133–29.378	0.000^a^
CHD	0.539	0.198–1.469	0.227
CHF	0.464	0.429–10.845	0.351
LA	5.266	1.592–17.419	0.006^b^
HT	5.290	0.529–52.844	0.156

a and b have statistical significance

## Conclusion

LA is associated with HT rather sICH and the prognosis at 3 months in elderly patients aged ≥ 60 years following IV rtPA in the Huairou District of Beijing. The severe LA may increase the risk of HT and the worse outcome at 3 months after thrombolytic therapy.

## Discussions

LA was first proposed by Hanchski in 1987 [12], and it was mainly described by imaging: low white matter density on brain CT, and high white matter signal on MRI T2 and Flair images. A previous study suggested that LA was associated with cognitive impairment/dementia and stroke risk [13]. With the increasing awareness of super-acute IV rtPA for stroke, we are confronting more and more risks for this therapy. Whether LA increases the risk of haemorrhage after thrombolysis is one of these inevitably realistic problems. As a common type of cerebral small vessel disease [14], LA incidence increases with age, and some studies suggest that it increases 2.4-fold every 10 years [9]. As the human lifespan continues to increase, the elderly may become a large proportion of patients receiving IV rtPA. For these populations, we should carefully wield the double-edged sword of thrombolytic treatment to provide the most suitable treatment strategies, improve their quality of life and reduce their social and financial burdens.

Most researches on LA and haemorrhagic transformation after thrombolysis has essentially arrived at a unanimous conclusion, which is that severe LA increases the occurrence of HT or sICH after thrombolysis [3, 15–17]. A similar result was observed from a meta-analysis in 2017 [18]. However, a few studies have reported contradictory results [5, 19]. Relevant research is extremely scarce in China, even in Asians, and such data on the elderly have rarely been reported. Yang et al. [20] obtained a negative correlation between LA and sICH, and their study was based on adults from Taiwan published in 2018. Is there a relationship between LA and sICH or HT in the Chinese population? What is the outcome for the growing proportion of elderly individuals? We carried out this study to answer these questions.

Our results showed that severe white matter lesions in elderly individuals aged ≥ 60 years increased the risk of HT but not sICH after IV rtPA and the latter accounting for a 9.6% incidence among all our patients. The incidence of sICH was higher than the 6.4% from NINDS [2] and the 7.2% from Alteplase Thrombolysis for Acute Non-interventional Therapy in Ischemic Stroke (ATLANTIS) [21], is similar to the 8.8% from ECASSII [10] and is slightly lower than the 10.8% from Prolyse in Acute Cerebral Thromboembolism-II (PROACTII) [22]. The reason for the higher proportion compared with the NINDS and ATLANTIS studies lies in the difference in the selected populations. The NINDS study only contained a few elderly people, and the ATLANTIS study excluded patients over 80 years; in contrast, our study focused on all elderly patients aged ≥ 60 years, among whom the oldest age was 96 years. A previous study indicated that the risk of haemorrhage following IV rtPA increased with age [23]. Certainly, the rate of sICH in the Asian population might actually be higher than that in Western countries [24]. Prior research has revealed that the severity of stroke increases the risk ratio of haemorrhage after thrombolysis [25]. The stroke severity observed in the PROACTII study was greater than ours; the average NIHSS score of the PROACTII study was 17, while our average score was approximately 11. This may account for why our sICH incidence was lower than theirs. Our results about the relationship of LA and HT were consistent with other existing studies [26–28], but the proportion of severe LA (35.9%) from France [28] was higher than ours (26.4%). There are two explanations for this difference: first, their sample consisted of an older population (aged over 75 years) than ours, and second, their imaging assessments were based on MRI, through which the detection rate of LA may be higher than our based on brain CT because of sensitivity [29]. Meanwhile we tried our best to adopt a uniform LA score system (mVSS) [11, 20], the results about the correlation of LA and sICH were accordant to the data from Taiwan in China [20]. Although all eligible adults presenting with acute stroke and receiving IV rtPA accepted in their research were younger (mean age approximately 67 years old) than ours (mean age approximately 73 years old), the final result was the same. Our correlation analysis simultaneously suggested that the severity of stroke and CHF in elderly patients were related to HT, but after binary regression analysis, only LA and NIHSS became the factors affecting the outcome. Therefore, the present study indicated that LA and severity of stroke in elderly patients were likely to influence HT independent of other risks after IV rtPA. We further analysed the relationship between LA and sICH, and the results were not related (*P* > 0.05). In summary we should pay more attention to and carefully evaluate patients before IV rtPA and actively adopted this treatment confronting elderly individuals with LA, including severe LA.

Plenty of researches had reported that LA is related to cerebral ischaemia, insufficient perfusion [30], venous collagenosis [31], genetic factors [32] and increased permeability of the blood-brain barrier (BBB) [33]. Animal experiments have shown that intravenous application of alteplase aggravated the destruction of the BBB in a suture occlusion model in rats [34]. These proofs may be the theoretical basis for the finding that patients with ACI accompanied by LA were more prone to HT after IV rtPA.

Our data also showed that LA was associated with adverse prognosis at 3 months after IV rtPA in elderly. This result is compatible with the conclusions of Western studies [27, 35]. The meta-analysis [27] suggested that LA doubled the incidence of poor outcomes at 3 months after IV rtPA. Our results on prognosis following thrombolytic treatment were consistent with those from Taiwan in China [20] and the outcomes reported in a Korean population [3]. White matter lesions impair the neuronal network between brain cells and synapses and ultimately affect the 3 months of functional recovery, which may be the reason for the effect of LA on bad prognosis [36].

Our study is a prospective observational study, and the subject population consisted of elderly individuals aged ≥ 60 years. At present, there are few similar studies on the relationship between LA, HT and prognosis at 3 months after IV rtPA in the elderly population, which is the greatest advantage of this study. Meanwhile, we strictly guaranteed the quality and authenticity of the data through measures such as blinded analyses, which included blindness between testers and subjects and between individual testers.

However, this study also has some shortcomings. First, we conducted only a single-centre study, and we had a small sample size that included 6 loss cases, which might have an impact on the outcome to some extent. Therefore, it is necessary to conduct additional, multi-centre, large-sample studies to provide additional evidence for our conclusions. Our hospital is the only stroke medical centre with thrombolytic qualification in the Huairou District of Beijing; thus, our data were integrated, which may reduce selective bias from patients to the maximum extent. Second, we used a brain CT scan to assess LA, which is less sensitive to head MRI and might cause underestimation of LA. However, CT scan is simpler and easier to perform, which is more suitable for elderly patients with severe illness or metal implants who cannot submit to head MRI examination. Thus, our examination would not only lessen sample selective bias for the above reasons but also greatly minimize the time delay for thrombolytic therapy because of its simplicity and practicality. Last, our study was observational one, and we need more convincing evidence from further RCT research.

In summary, we concluded that LA is associated with HT rather sICH and poor clinical prognosis after IV rtPA in elderly patients aged ≥ 60 years with ACI in the Huairou District of Beijing: the severe LA may increase the risk of HT and the worse prognosis at 3 months after thrombolytic treatment. When implementing IV rtPA, we should pay more attention to the complications of HT for elderly acute stroke patients with LA, but this should not affect clinicians’ treatment decisions. At the same time, we should strive to attain preliminary and accurate prediction of the prognosis for this population.
